# Factors affecting major depression in Iran: a mixed-method study

**DOI:** 10.1186/s41043-024-00571-x

**Published:** 2024-05-27

**Authors:** Zarrintaj Hosseinzadeh-Shanjani, Rahim Khodayari-Zarnaq, Mohammad Farough Khosravi, Morteza Arab-Zozani, Gisoo Alizadeh

**Affiliations:** 1 Social Determinants of Health Research Center, Zanjan University of Medical Sciences, Zanjan, Iran; 2 Department of Health Policy and Management, School of Management and Medical Informatics, Tabriz University of Medical Sciences, Tabriz, Iran; 3 Department of Health economics and management, School of Public Health, Tehran University of Medical Sciences, Tehran, Iran; 4 Social Determinants of Health Research Center, Birjand University of Medical Sciences, Birjand, Iran

**Keywords:** Social factors, Economic factors, Environment, Political factors, Technology, Major Depression Disorder, Mixed-method study, Iran

## Abstract

Depression is a major cause of disability and, if left untreated, can increase the risk of suicide. Evidence on the determinants of depression is incomplete, making it challenging to interpret results across studies. This study aims to identify the social, economic, environmental, political, and technological factors influencing the great recession in Iran. The study was conducted in two parts. The first step involved a literature review to identify the factors, using PubMed, Scopus, and Web of Science for the search. The reference lists of all identified articles were reviewed to find relevant studies, and the extracted information was summarized and reported descriptively. The second steps involved compiling and consulting 14 experts from different fields, using a framework analysis method. Twenty-four articles were used as primary sources of information, and a total of 28 factors were found to exist. After removing duplicates and related factors, 19 of these were subsequently declared as factors, resulting in a total of 36 determinants being identified. Most of these factors belong to the social category. The health policies implemented have a significant impact on disease risk factors and ultimately their occurrence. Political decisions and policy-making processes play a crucial role in all areas, particularly in addressing disease risk factors. Severe depression can disrupt all aspects of the healthcare system, underscoring the importance of access to care. Policies concerning physical education, transportation, nutrition, employment, green spaces, recreational facilities, and tobacco are vital in this context. The influence of health policies on disease risk factors and disease occurrence is profound. Severe depression can have far-reaching effects on the healthcare system, emphasizing the critical need for access to care. The formulation of policies to combat depression must be thoroughly evaluated in terms of economic, political, social, technological, and environmental factors. The findings suggest that addressing social inequalities and emphasizing the role of political action, as highlighted by the social determinants of health, should be top priorities in addressing depression. Efforts to prevent depression should incorporate ecological approaches that consider the impact of the socioeconomic environment on depressive symptoms.

**Supplementary Information:**

The online version contains supplementary material available at 10.1186/s41043-024-00571-x.

## Introduction

Mental health plays an important role in the dynamic and efficient functioning of any society. In the growth and development of cultural, social, and economic societies, the mental health of the society is very decisive [1, 2]. Mental health is a function of various social and cultural factors. Meanwhile, depression is one of the most common mental illnesses in which social factors play an effective role. Depression occurs in older adults, similar to younger people due to socio-psychological and biological factors [3]. Risk factors for depression include social isolation, marital status, divorce or separation, low socioeconomic status, debilitating comorbidities, insomnia, and functional and cognitive disorders [4]. Studies have shown that the prevalence of mental disorders such as depression, and anxiety is related to factors such as unemployment, low income, limited education, stressful working conditions, gender discrimination, and unhealthy lifestyles [5, 6]. Feelings of sadness and lack of interest in daily activities are defining features of major depressive disorder (MDD). Depressed people have cognitive impairment with problems of indifference, difficulty expressing opinions, and feelings of hopelessness [7]. Factors that are effective in the occurrence of depression include lack of social support, living in rural areas, suffering from chronic diseases, smoking, and alcohol and drug abuse [8]. The Marmot Report, the WHO, and Compton et al. all address the same issue: the most impoverished individuals in society are facing significant challenges, particularly due to their financial circumstances. As a result, there are disparities in mental health outcomes [9–11].

350 million people in the whole world suffer from depression, and this disease has a higher prevalence in low- and middle-income countries [12]. A higher prevalence of depression is associated with lower socioeconomic status (SES) [13]. SES affects the occurrence of depression and several studies confirm the negative relationship between SES and depression [14]. . A study in Spain showed that MDD is strongly associated with socio-economic disadvantage in both genders [15]. The disease, which affects the quality of life, brain function, work status, and productivity, is one of the most serious in the world [16, 17]. The number of adults with MDD in the United States increased 12.9% from 15.5 to 17.5 million between 2010 and 2018, while the proportion of adults aged 18–34 with MDD increased from 34.6 to 47.5%. Over the same period, an incremental economic burden on adults with MDD rose by 37.9% to $326.2 billion from $236.6 billion in 2020 values [18]. Considering the importance of depression and the need for efficient prevention and treatment of this disease, most social factors are effective in the occurrence of this disease, and from that point, effective policy-making in the field of prevention or treatment of diseases is possible in the shadow of accurate and scientific information.

This research aimed to thoroughly examine the causes of Iran’s severe depression, including society, economy, technology, and environment. Since most research has focused on specific risk factors in one country or region, this study aims to systematically review existing literature on major depression in Iran and present the related factors. The findings from the study could assist policymakers and healthcare providers in creating successful interventions for major depressive disorder when making decisions.

## Methods

### Study design

This mixed-method study was conducted over two sections. First, social, economic, technological, and environmental factors had been identified through a literature review. Then, Experts participated in completing and promoting the list of identified factors.

### Step 1

We searched PubMed, Scopus, and Web of Science. Moreover, the reference list of all those articles which have been identified was examined to determine relevant studies. We limited our search to those articles published in English and made a final search on June 2022. The search has not been subject to a time limit. In parallel, a preliminary search was carried out and keywords were obtained from the same studies. The following keywords were searched: Depressive Disorder, Major, Risk Factors, Factor, social, technological, environmental, economic, socio-cultural and ecological. We use Boolean Operators (+, AND) to combine search terms in library databases.

The study was composed of previously published studies on MDD and those which have been described as one or more determinants of MDD. Conference paper abstracts (where full analysis was not available), case reports, and Studies published only as an abstract were excluded. Two referees independently checked the titles and subheadings of each article. Full text has been collected and reviewed by reviewers independently to determine whether a study should be included or excluded. The disagreement to study eligibility was resolved either through negotiations between the reviewer or by a third party.

The information extracted was descriptively summarized and reported. The author, country, publication year, study description, objective, and results were added to the data obtained. Two steps were taken in the analysis. First, the characteristics of these studies were summarized as follows: author, country, publication year, study description, objectives, and results. A complete review of the relevant studies was carried out, and necessary data were obtained. Second, the identification factors have been based on specific study results that focus specifically on key determinant factors of MDD. Arranging studies, reading titles and abstracts as well as identifying duplicates is carried out using Endnote X20.

### Step 2

Experts related to MDD have been enrolled in the study population. Interviews took place with 14 experts in different areas such as health policy, social services management, psychologists, and psychiatrists. The experts were carefully selected and those who have the most knowledge and experience in their field of study are chosen. At least 2 relevant articles and a minimum of 5 years of experience were considered as criteria for selection. A purposeful sampling method has been used and will continue until saturation. A guide for interviews based upon the review result and a semi structured interview was carried out. (Appendix 1: interview guide) The interviews were done by two researchers from the Research Team: a lead investigator with prior experience of conducting research based on qualitative interviews and one PhD student. To coordinate and conduct interviews, student researchers have been trained in skills, attributes, practices, and specific project tools. The interviews were held virtually on the platform chosen by the participant (mostly via Skype). The interviews were conducted in a time frame that was convenient for the interviewee. Interviews were recorded with consent. It has been tried to participate in the study from different regions of the country. In addition, a written agreement was obtained from participants so that the interview could be recorded. Interviews have been given back to the participants for evaluation and approval at the end of each interview to increase consistency in the study. Moreover, interviews were transcribed immediately and the findings were analyzed using the framework analysis. After that, the transcribed text was reworded repeatedly and appropriate codes were subsequently obtained. The analyses of the items in the Final List have been carried out based on the SOEP framework: social, economic, political, environmental and technological.

To get the study to be precise and valid, criteria of credibility, reliability as well as data transferability have been examined. The criterion for data integrity has been deemed to be credibility, conformity, dependency, and transferability [19, 20]. (Barnett-Page & Thomas, 2009; Ward, Furber, Tierney, & Swallow, 2013). To check for accuracy and resonance, the content of the interview was returned to the participants. The experts who have a good knowledge of this field were deliberately selected. If there is a difference of opinions, the final decision will be announced by a third party.

## Result

In 3 databases, 5934 articles were found during the initial search. Then 978 original articles from the remaining 4956 have been screened using title and abstracts after being cleaned for duplicate or without full text. To allow a deliberate review, the papers which meet all eligibility criteria have been selected. In addition, 24 articles were used as the main source of information on factors after they had been read in full text. (Appendix 2: Extraction table) In Table 1, the Individual characteristics of research participants were reported.

**Table 1:** Individual characteristics of research participants

Expert’s code	Gender		Age	Education	Expert
	Male	Female			
1	*		69	Ph.D.	health policy
2	*		58	Master	health policy
3		*	46	Ph.D.	health policy
4	*		49	M.D	Psychiatrist
5	*		47	Ph.D.	Psychologist
6	*		45	Ph.D.	social services management
7		*	35	M.D	Psychiatrist
8	*		60	M.D	Psychiatrist
9	*		62	M.D	Psychiatrist
10		*	54	Master	social services management
11	*		42	Ph.D.	Psychologist
12	*		38	Ph.D.	social services management
13		*	36	M.D	Psychologist
14		*	32	M.D	Psychologist

Based on the studies that were included, 28 factors were found to be in existence and 19 of them have subsequently been declared as factors following removal of duplicates and related factors. Religiosity, low physical activity, overweight, family dynamics, and family history of psychiatric disorders are social factors affecting depression, which are identified in the review. Childhood and events related to it are mentioned in the studies. Work-related stress and one’s surroundings play a crucial role. Engaging in risky behaviors and using drugs is connected to experiencing depression. In the following, promoted to a 36-item list adding the suggested factors by the experts (Table 2).

**Table 2:** Factors identified in STEEP framework

Category	Factor	Determinant
**Social**	Socio-demographic	Religiosity
		low physical activity
		Overweight
		Family dynamics
		Family history of psychiatric disorders
	Violence	Early abuse
		Partner violence
	Drug and Smoking	Tobacco consumption
		Cannabis use
	Occupation	Job strain
		Job insecurity
		Job stress (work demand and decision latitude)
	childhood experience	childhood maltreatment
		Parental warmth
	Social inequalities	discrimination
		social support
	Eating habits	heavy episodic drinking
		healthy dietary pattern
	Others social issues	cancers
		Loneliness
		stressful life events
**Economical**	Income	Annual family income
		Income inequality
	economic depression	recession
		unemployment
		Inflation
**Technological**	Rapid technology	Industrialization
		Urbanization
**Environmental**	Environmental pollution	Air pollution
		Noise pollution
	Living environments	Built environment
		Traffic
		Access to green space
	Disaster	Natural disaster
**Political**	Political crises	Sanction
		The possibility of war

Most determinants belonged to the social category, whereas the economic and technological categories consisted of the least number of determinants. In the social category, determinants were related to socio-demographics, Violence, Drug and Smoking, Occupation, childhood experience, social inequalities, and Eating habits. Moreover, the emphasis was more on the impact of family and childhood period. “*Family is very important in the treatment and prevention of mental illnesses, especially depression*.” (P1) “*Most of the factors start from families and get worse*.” (P10). The financial crisis that directly affects household income, which is increasing in Iran, was one of the major issues that were emphasized in the interviews. “*Financial issues are very worrying. In most families, only one person works and pays for the rest… This means a crisis*” (P3) Furthermore, this may lead to the development of MDDs due to inequalities and unequal distribution of revenues at individual and community level. The advancement of technology has caused industrialization and urbanization, these factors also cause environmental pollution. It is important to ensure that air pollution and the composition of the living environment are taken into account for access to recreational facilities and physical activity.*” There has been so much migration to the cities that traffic and air pollution have become very severe”* (P5).

It is important to work on the biological and psychological aspects of health as well as on the social roots of disease, which broadens planners’ perspectives on possible interventions. The results of the present study can guide policymakers to target the main cause of diseases and better evaluate the impact of previous health interventions at the community level. Policymakers should also consider social aspects in formulating health policies, and this may lead to more effective policies and fewer errors in prioritization and interventions. Doctors need to think of medicine as a social issue to help patients. Various elements within society can trigger and intensify severe depression. Various factors are interconnected and influence one another. For instance, political and social issues are affected by economic crises, and the reverse is also true.

**Fig. 1:**
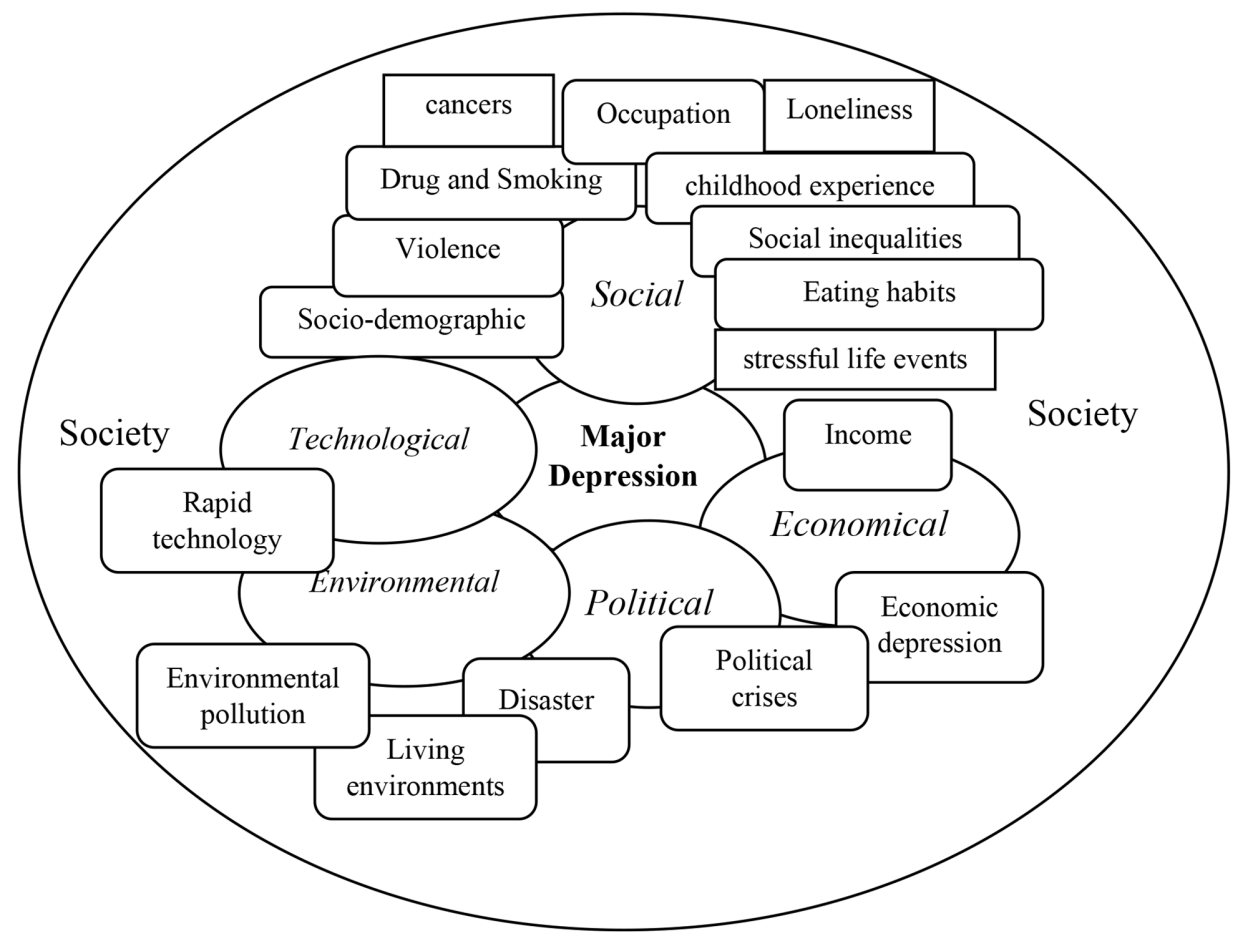
Conceptual relationships between categories, factors, determinants, and major depression

In Fig. 1, the conceptual relationships between categories, factors, and determinants are presented.

## Discussion

A total of 36 determining factors were identified. Most of these factors belonged to the social category. Adopted health policies greatly affect the risk factors of diseases and ultimately the occurrence of diseases. Political decisions and policy-making processes are effective in all fields, especially in the field of disease risk factors. In this regard, major depression can affect all functions of the health system, making access to care very important. Policies related to physical education, transportation, food, work, green space, recreational facilities and tobacco are very important in this context.

Depressive disorder is etiologically complex with multiple risk factors involving biological, environmental, and psychological factors [21, 22]. Several social determinants of health (SDOH) have been associated with the onset of major depressive disorder [23]. Depression is associated with social risk factors, disorders, and poor social functioning [24]. A research project was undertaken to explore how socioeconomic surroundings impact depressive symptoms in adults from Taiwan, using multilevel analysis. The results showed that two social environments and two economic characteristics have a significant relationship with depression symptoms. However, the effect of these factors was different according to gender and age group. Economic environments were critical for men and young adults aged 20–44, while social environments were significant for women and middle-aged and older adults [25].

The result of a review shows that the timing of events has significance when it comes to mental health. Early life is a critical period during the lifespan at which cognitive processes develop. Exposure to harmful determinants, such as stress, during this period can place an individual on a trajectory of depression in adulthood or later life. When an individual is exposed to harmful determinants during critical periods and is also genetically predisposed to depression, the risk for the disorder can be compounded. This is why aspects such as the lifecourse perspective and gene–environment interactions need to be taken into account. Insight into this can also help to refine targeted interventions [26]. Adverse childhood experiences can affect emotional and psychological development and increase susceptibility to mental health issues such as major depression and post-traumatic stress disorder (PTSD) [27].

Economic recession has various effects on mental health at an individual, family, and epidemiological level [28]. Evidence was consistent that economic downturns and mediators such as unemployment, reduced income, and unmanageable debt were significantly associated with poor mental well-being, increased rates of common mental disorders, substance-related disorders, and suicidal behaviors [29]. For example, the results of studies show in the wake of a global economic recession to the COVID-19 pandemic, depression increased [30, 31]. The impact of the economic crisis on the mental health of the population is pervasive. The primary prevention and early identification of major depression should be a priority for services and specialists [32].

Urbanization is increasing globally, and is associated with stress and increased mental health risks, including for depression [33]. Evidence is beginning to emerge that rates of psychiatric disorders such as anxiety and depression are rising in younger generations partly because of inter-generational inequalities where younger people feel that they are being left behind on several material parameters in addition to other factors such as urbanization and industrialization [34]. In the countries of Southeast Asia which have experienced rapid industrialization, there is a more frequent occurrence of occupational diseases and injuries [35].

The findings of a study show that residential space and block density may play a role in causing depression. A population based approach to reducing depression burden can be a combination of Integrated Mental Health and Housing Policies which promote psychological capital in cities, for example optimal health densities at home or building level [36]. The urban environment has a potential for improving air and water quality, reducing floods, increasing physical and mental wellbeing as well as promoting social and cultural good health. However, the true value of ecosystem services for cities around the globe is not yet known, in particular at a time when they are subject to an ever widening range of societal, ecological and technological contexts [37].

Political, economic, social, environmental and technological factors all over the world affect the prevalence of mental illnesses, especially major depression, but in Iran, the presence of some of these factors in bold, such as economic and political crises, certainly has a greater impact that should be studied.

The diversity of experts who take part in the study is one of its main strengths. The opinions of experts from a variety of fields have been considered as part of the study and this has further strengthened the validity of the results. Another point of strength is to be a multimethod study. The experts who took part in that study were from Iran, and this may limit its generalizability. Furthermore, the assessment in the initial phase was incomplete or systematically insufficient and it could be regarded as a further limitation.

## Conclusions

The creation of strategies to address schizophrenia needs to be thoroughly examined considering economic, political, social, technological, and environmental factors. Results show that when dealing with depression, it is crucial to prioritize addressing social inequality and policy interventions as highlighted by social determinants of health. Efforts to prevent depression should include ecological strategies that consider the impact of socioeconomic environments on depressive symptoms.

### Electronic supplementary material

Below is the link to the electronic supplementary material.

Supplementary Material 1

## Data Availability

Data is provided by sending an email to a corresponding author.
