# Supplementary material for: Factors affecting major depression in Iran: a mixed-method study
Source: J Health Popul Nutr. 2024 May 27;43:73. doi: 10.1186/s41043-024-00571-x (PMC11131193; doi:10.1186/s41043-024-00571-x)
Supplement: Supplementary file 1 — Supplementary Material 1 [file 41043_2024_571_MOESM1_ESM.docx]

Appendix

(Appendix 1: Interview guide)

**Explain the objectives of the study**

**Get written permission to participate in the study**

**Get permission to record audio**

Name of the interviewee:

Organizational position of the interviewee:

Relevant organization to be interviewed:

Date and time of the interview:

Interview location:

Work experience of the interviewee related to depression:

**Question**

The following table is obtained from the results of the review. In your opinion, what factors in causing major depression in Iran can be added to the list below?

| **Factor** | **Determinant** |
| --- | --- |
| Socio-demographic | religiosity |
|  | low physical activity |
|  | overweight |
|  | family dynamics |
|  | family history of psychiatric disorders |
| Violence | Early abuse |
|  | Partner violence |
| Drug and Smoking | Tobacco consumption |
|  | cannabis use |
| Occupation | Job stress (work demand and decision latitude) |
| childhood experience | childhood maltreatment |
|  | Parental warmth |
| Social inequalities | discrimination |
|  | social support |
| Eating habits | heavy episodic drinking |
|  | healthy dietary pattern |
| Others | cancers |
|  | stressful life events |
| Disaster | Natural disaster |

(Appendix 2: Extraction table)

| No | **Author, Country, publish year** | **Study Description** | **Objective** | **Result** |
| --- | --- | --- | --- | --- |
| 1 | Y. S. Zhang nad et al. China, 2020 (32) | Multistage, stratified, random sampling was administered between April and August 2016. A total of 3911 participants were enrolled. | This study examined the 1-month and lifetime prevalence of MDD and its socio-demographic correlates in older adults living in Hebei province, an agricultural area of China. | female gender [P < 0.001, adjusted odds ratio (aOR) = 2.6, 95%CI: 1.53-4.53], presence of comorbid major medical conditions (P < 0.001, aOR = 4.8, 95%CI: 2.17-10.39) and family history of psychiatric disorders (P = 0.013, aOR = 3.4, 95%CI: 1.30-8.96) were independently and significantly associated with higher odds of MDD. |
| 2 | G. C. Medeiros and et al. USA. 2020 (33) | We analyzed the baseline data of 663 individuals from the CO-MED study. | This study examined: 1) the prevalence of childhood maltreatment (CMT) in individuals with chronic and/or recurrent depression, 2) the association between CMT and depressive symptoms, 3) the link between CMT and worse clinical presentation of depression, 4) the effects of accumulation of different types of CMT, and 5) the relationship between the age at CMT and depression. | Half of the sample (n = 331) reported CMT. Those with CMT had higher rates of panic/phobic, cognitive and anhedonic symptoms than those without CMT. All individual types of maltreatment were associated with a poorer clinical presentation including: 1) earlier MDD onset; 2) more severe MDD, 3) more suiccidality, 4) worse quality of life, and functioning, and 5) more psychiatric comorbidities. |
| 3 | Daiki Kobayashi and et al. Japan. 2020(34) | A longitudinal study was conducted in a large hospital in Tokyo, Japan, from 2005 to 2018. 67 723 adult participants | The objective was to evaluate the association between religiosity and subsequent depression in a multireligious population. | extremely religious group (OR, 1.51; 95% confidence interval [CI], 1.28-1.78) and the moderately religious group (OR, 1.30; 95% CI, 1.14-1.49) were statistically associated with increased development of MDD compared to the not-religious-at-all group. Those who had increased their religiosity from baseline had statistically lower development of MDD (OR, 0.85; 95% CI, 0.75-0.97) compared to those who remained in the same degree of religiosity from baseline. |
| 4 | Y. C. E. Chung and et al. Taiwan.2019(35) | We recruited 36 MDD patients and 37 healthy controls for 16S rRNA gene sequencing. | The current study aimed to identify microbiota targets for major depressive disorder (MDD) and mood-related traits in Taiwanese samples, while taking into account the influence of dietary patterns. | At genus level, Bifidobacterium (7%) and Blautia (8%) had relatively high abundance among MDD patients, while Prevotella (16%) had high abundance in controls. Holdemania exhibited moderate correlation with anxiety (r = 0.65) and perceived stress level (r = 0.49) mainly in MDD patients but not controls. |
| 5 | R. L. Rohde and et al. USA. 2018(36) | 71 541 cases of HNC using a national dataset, the Nationwide Inpatient Sample, from 2008 to 2013. | This study explored the prevalence of and sociodemographic and clinical factors associated with depression, among patients with head and neck cancer. | For every unit increase in comorbidities, odds of depression increased by 20% (aOR = 1.20; 95% CI, 1.19-1.23).  Depression odds vary depending on HNC anatomic site, and one in four patients with laryngeal cancer may be depressed. |
| 6 | A. Y. Kudinova and et al. Cauca. 2018 (37) | We recruited 458 parents and their children between the ages of 7-11 from the community | The current study examined whether child emotion regulation, specifically, the use of cognitive reappraisal and suppression, moderated the link between parent and child depression. | These findings suggest that, among children with a history of parent depression, children's use of cognitive reappraisal may influence their own risk for developing depression and highlights the potential utility of early interventions that focus on improving the use of emotion regulation strategies like cognitive reappraisal among children of depressed parents. |
| 7 | O. Santesteban-Echarri and et al. USA. 2017(38) | Boricua Youth Study participants, Puerto Rican children 5 to 13 years of age at Wave 1 living in the South Bronx (New York) (SB) and San Juan and Canguas (PR) (n = 2,491), were followed for three consecutive years. | We prospectively examined the association between Parental warmth and child psychiatric disorders (anxiety, major depression disorder, ADHD, disruptive behavior disorders) | Higher levels of PW were related to lower odds of child anxiety and major depressive disorder over time (OR = 0.69[0.60; 0.79]; 0.49[0.41; 0.58], respectively). |
| 8 | B. Park and et al. South Korea. 2017(39) | This was a 1-year cross-sectional study using a national patient sample provided by the South Korean National Health Insurance in 2011. | we explored the prevalence rates of depression among the ten most prevalent cancers in South Korea using a national patient sample. | The prevalence of depression varied according to cancer types. Patients with lung cancer were the most prone to experience depression. |
| 9 | C. J. Bell and et al. New Zealand. 2017(40) | (n=495)- | The aims of this study were to do this by examining the roles of peri-traumatic stress and distress due to lingering disaster-related disruption in explaining linkages between disaster exposure and major depressive disorder symptoms among a cohort exposed to the 2010-2011 Canterbury (New Zealand) earthquakes. | The associations between earthquake exposure and major depression were explained largely by the experience of peri-traumatic stress during the earthquakes (=0.180, p<0.01). |
| 10 | C. Rahe and et al, Germany. 2016(41) | A sample of 823 patients with MDD and 597 non-depressed controls was examined | The aim of this study was to examine associations of [major depressive disorder](https://www.sciencedirect.com/topics/medicine-and-dentistry/major-depressive-episode) (MDD), its distinct subtypes, and symptom severity with the individual lifestyle factors smoking, diet quality, physical activity, and [body mass index](https://www.sciencedirect.com/topics/medicine-and-dentistry/body-mass-index) as well as with a combined lifestyle index measuring the co-occurrence of these lifestyle factors. | After adjustment, MDD was significantly associated with smoking, low physical activity, and overweight. Likewise, MDD was significantly related to the overall lifestyle index. |
| 11 | P. Pedrelli and et al. England. 2016(42) | 61,561 (65.3% female) | This study examined the association between MDD and HED in this population, the effect of gender on this association, and whether comorbid MDD and heavy alcohol use are associated with higher rates of mental health treatment engagement. | Students with MDD reported more frequent HED and higher pBAC than did students without MDD; this was especially true for female students. |
| 12 | S. Park and et al. Asian countries. 2015 (43) | A total of 507 outpatients with MDD were recruited in China (n = 114), South Korea (n = 101), Malaysia (n = 90), Thailand (n = 103) and Taiwan (n = 99). | We aimed to compare types of stressful life events associated with the onset of depressive episodes in patients with major depressive disorder (MDD) in five Asian countries. | The type of stressful life event that preceded the onset of a depressive episode differed between patients in China and Taiwan and those in South Korea, Malaysia and Thailand. Patients in China and Taiwan were less likely to report interpersonal relationship problems and occupational/financial problems than patients in South Korea, Malaysia and Thailand. |
| 13 | A. Schulz and et al, Germany. 2014 (44) | Two thousand and forty-six subjects aged 29-89 | This study aims at investigating the influence of dispositional resilience on this relationship. | The findings support the clinical assumption that resilient subjects may be partly protected against the detrimental long-term effects of child abuse and neglect. |
| 14 | J. J. J. Schuch and et al. Netherland. 2014 (45) | 1115 participants (364 men, 751 women, mean age 41 years) | This study aims to identify gender differences in psychopathology, treatment, and public health consequences in patients with MDD. | Main gender differences in the clinical presentation of MDD concerned a younger age of onset, higher anxiety and lower alcohol use comorbidity and higher prevalence of atypical depression in women. These differences were accompanied by differences in health care use. |
| 15 | B. Rashidkhani and et al. Iran. 2013 (46) | 45 women with major depression and 90 patients with no mental disorder participated. | investigating the association of dietary patterns and anthropometric status with major depression in adult women living in Tabriz. | These results suggest that the healthy dietary pattern is significantly associated with lower odds of major depression in adult women. |
| 16 | J. A. Pasco And et al. Australia.2012(47) | Eighteen women who developed de novo MDD were classified as cases; there were 298 controls. | We aimed to determine if low levels of dietary selenium are associated with an increased risk for de novo major depressive disorder (MDD). | These data suggest that lower dietary selenium intakes are associated with an increased risk of subsequent de novo MDD. |
| 17 | D. H. Chae and et al. Asian Americans. 2012 (48) | Asian American adults (N = 2095) were used to examine associations between discrimination, negative interactions with relatives, family support, and 12-month major depressive disorder (MDD). | This study examined whether discrimination and family dynamics are associated with depression in this population. | Results suggest that discrimination may have negative mental health implications, and also point to the importance of family relationships for depression among Asian Americans. |
| 18 | A. Bulloch and et al. Canada. *2012 (49)* | 17,276 household residents | We evaluated the incidence of MDE in relation to different patterns of alcohol use, and examined the incidence of alcohol misuse in respondents with and without MDE. | Of direct clinical significance is the bidirectional relationship between alcohol dependence and MDE. |
| 19 | M. Tao And et al. China. 2011(50) | 60% of controls and 72% of cases reported at least one lifetime SLE. | We examined 1970 Chinese women with recurrent MDD, and compared the prevalence of 16 SLEs in matched controls. | More severe stressful life events are more strongly associated with MDD. These results support the involvement of psychosocial adversity in the etiology of MDD in China. |
| 20 | O. S. Schwartz and et al. Australia. 2011(51) | 159 adolescents (aged 11-13 years) with no history of MDD | The aim of this study, therefore, was to indentify which patterns of maternal response to their children’s dysphoric and aggressive behaviour (i.e., either the elicitation or suppression of maternal dysphoria or aggression) were predictive of the onset of MDD. | Mothers' responses to adolescents' aggressive and dysphoric behaviours may differentially influence the risk of MDD onset for adolescents over time. |
| 21 | C. S. Widom and et al. USA. 2007 (52) | Children with substantiated cases of physical and sexual abuse and neglect (before the age of 11 years) from January 1, 1967, to December 31, 1971 (n = 676) were matched based on age, race, sex, and approximate family social class with a group of non-abused and non-neglected children (n = 520) and followed up into young adulthood (mean age, 28.7 years). | To determine whether abused and neglected children were at elevated risk of major depressive disorder (MDD) and psychiatric comorbidity, compared with matched control subjects, when followed up into young adulthood. | Child abuse and neglect were associated with an increased risk for current MDD (odds ratio [OR], 1.51; 95% confidence interval [CI], 1.06-2.14; P< or=.05) in young adulthood. Children who were physically abused (OR, 1.59; 95% CI, 1.00-2.52; P< or =.05) or experienced multiple types of abuse (OR, 1.75; 95% CI, 1.01-3.02; P< or =.05) were at increased risk of lifetime MDD, whereas neglect increased risk for current MDD (OR, 1.59; 95% CI, 1.10-2.29; P<.01). Childhood sexual abuse was not associated with elevated risk of MDD. Kaplan-Meier age-of-onset curves (log-rank statistic, 4.03; df = 1; P=.04) showed earlier onset of MDD for abused and neglected children compared with controls. Among those with MDD, comorbidity was higher for abused and neglected individuals than for controls. |
| 22 | M. van Laar and et al. Netherlands. 2007 (53) | The analysis was carried out on 3881 people who had no life-time mood disorders and on 3854 people who had no life-time anxiety disorders at baseline. | To investigate whether cannabis use predicted the first incidence of mood and anxiety disorders in adults during a 3-year follow-up period. | any use of cannabis at baseline predicted a modest increase in the risk of a first major depression (odds ratio 1.62; 95% confidence interval 1.06-2.48) |
| 23 | M. Melchior and et al. Denmark. 2007(54) | a 1972-1973 longitudinal birth cohort assessed most recently in 2004-2005, at age 32 (n = 972, 96 % of 10 15 cohort members still alive). | We tested the influence of work stress on diagnosed depression and anxiety in young working adults. | Participants exposed to high psychological job demands (excessive workload, extreme time pressures) had a twofold risk of MDD or GAD compared to those with low job demands. |
| 24 | R. A. Brown, and et al. USA. 1996(55) | A representative sample of 1,709 adolescents (aged 14 through 18 years) was assessed by using semi-structured diagnostic interviews on two occasions, approximately 1 year apart. | To examine cross-sectionally and prospectively relationships of cigarette smoking with major depressive disorder (MDD), controlling for comorbidity in a community sample of adolescents. | The results suggest important relationships between cigarette smoking and psychiatric disorders among adolescents, particularly with regard to MDD, drug abuse/dependence, and disruptive behavior disorders. |
